# Cell Wall Invertase Inhibitor SlINVINH1 Acts as a Negative Regulator in Fruit Ripening of Tomato

**DOI:** 10.3390/plants15060942

**Published:** 2026-03-19

**Authors:** Siran Chen, Hongjian Wan, Jiaxiang Wei, Yonghua Liu, Jun Li

**Affiliations:** 1 School of Breeding and Multiplication (Sanya Institute of Breeding and Multiplication), Hainan University, Sanya 572022, China; 2 Hainan Institute of Zhejiang University, Sanya 572025, China; 3 School of Tropical Agriculture and Forestry (School of Agricultural and Rural Affairs, School of Rural Revitalization), Hainan University, Danzhou 571737, China; 4 Institute of Vegetables, Zhejiang Academy of Agricultural Sciences, Hangzhou 310021, China

**Keywords:** tomato fruit ripening, cell wall invertase inhibitor, sugar metabolism, CRISPR/Cas9, transcriptome

## Abstract

Sugar metabolism is an important factor in influencing fruit ripening, while the associated mechanism is not well understood. Cell wall invertase (CWIN) inhibitors play important roles in plant organ (such as fruit, seed, leave, tuber) development and stress resistance, as they are able to regulate CWIN activity through protein–protein interaction, affecting sugar levels in plants. Here, we report a novel role of one tomato CWIN inhibitor in regulating fruit ripening. Specifically, knockout of *SlINVINH1* gene via CRISPR/Cas9 technique accelerated the onset of fruit ripening process, along with the increase in CWIN activity and contents of sucrose, glucose, fructose and carotenoid and decrease in chlorophyll content in ripening fruits of the CR-*slinvinh1* mutants. Transcriptome analysis demonstrated that the differentially expressed genes (DEGs) in fruits of CR-*slinvinh1* were enriched in several biological pathways related to fruit ripening and/or sugar metabolism. The expression levels of invertase genes and inhibitor genes in CR-*slinvinh1* were consistent with the alterations of invertase activity and sugar levels. Moreover, the transcript levels of a set of pivotal ripening-related marker genes including the global ripening regulator gene *SlRIN* were increased in ripening fruits of *CR-slinvinh1*. This study provides novel insights into the regulatory network underlying tomato fruit ripening, as well as a new genetic strategy using CWIN inhibitor genes to simultaneously accelerate fruit ripening and increase fruit sweetness.

## 1. Introduction

The ripening of fleshy fruits is a complex biological process that involves a series of physiological and biochemical changes, influencing the color, aroma, texture, flavor, and other characteristics of the fruit [1,2]. Color transformation typically occurs during the fruit ripening process as chlorophyll breaks down, while pigments such as carotenoids (yellow/orange) or anthocyanins (red/purple) are synthesized and appear, causing the fruit to acquire its mature color [1,3]. The sugar content often increases during fruit ripening, as starch in the fruit normally is broken down into soluble sugars such as glucose and fructose, resulting in an increase in sweetness.

In higher plants, sucrose is the main sugar that is synthesized in photosynthetic leaves (source) and transported over long distance to sink organs such as the fruit, flower, seed [4,5]. Sucrose needs to be broken down into hexoses before it can be utilized by the plant sink organs for the synthesis of starch, cellulose, proteins required to promote plant growth and resist stress conditions. The hydrolysis of sucrose into monosaccharides in plants is mainly catalyzed by two types of enzymes, sucrose synthase and invertase [5,6,7]. Sucrose synthase reversibly cleaves sucrose into UDP-glucose and fructose in the presence of UDP, whereas invertase irreversibly hydrolyses sucrose into glucose and fructose. Based on the difference in subcellular localization, invertase is classified into three isoforms: cell wall invertase (CWIN), vacuolar invertase (VIN) and cytoplasmic invertase (CIN) [5,8,9,10].

Plant invertase genes are highly regulated from the transcriptional to posttranslational levels [4,5]. At the posttranslational level, invertase activity is typically modulated through protein–protein interaction by invertase inhibitors [11,12]. To date, invertase inhibitors have been identified for CWIN and VIN and not for CIN. Plant invertase inhibitors precisely regulate sugar distribution by inhibiting the enzyme activity of invertase, controlling sucrose breakdown into glucose and fructose. This impacts the source–sink relationships, organ development (like fruit, seed, and leaves), and stress responses, acting as a key link between sugar metabolism and plant growth, development, and adaptation [5,11,13,14,15].

Tomato (*Solanum lycopersicum* L.) has been selected as a model system for studying fruit ripening, and its fruit ripening is a highly coordinated developmental process involving the expression and regulation of thousands of genes [16,17]. To date, the molecular regulatory networks underlying the tomato fruit ripening process have been elaborately elucidated via comprehensive analyses of several related mutants including *ripening* *inhibitor* (*rin*), *non-ripening* (*nor*), etc. [2,18]. Consumers worldwide typically prefer sweeter fruits including tomatoes. Although CWIN inhibitors have been demonstrated to regulate the fruit sugar levels during fruit ripening via modulating the activity of CWIN, affecting fruit sweetness, whether they control other aspects of fruit ripening have rarely been reported. In the present study, we report a novel function of the CWIN inhibitor in fruit ripening process for the first time in plants. Specifically, we found the tomato CWIN inhibitor SlINVINH1 acts as a negative regulator towards the onset of fruit ripening. Our study provides novel insights into the regulatory network underlying tomato fruit ripening, as well as a new genetic strategy to increase the fruit sweetness and accelerate the fruit ripening process simultaneously.

## 2. Results

### 2.1. Knockout of SlINVINH1 Accelerated Tomato Fruit Ripening

**Figure 1:**
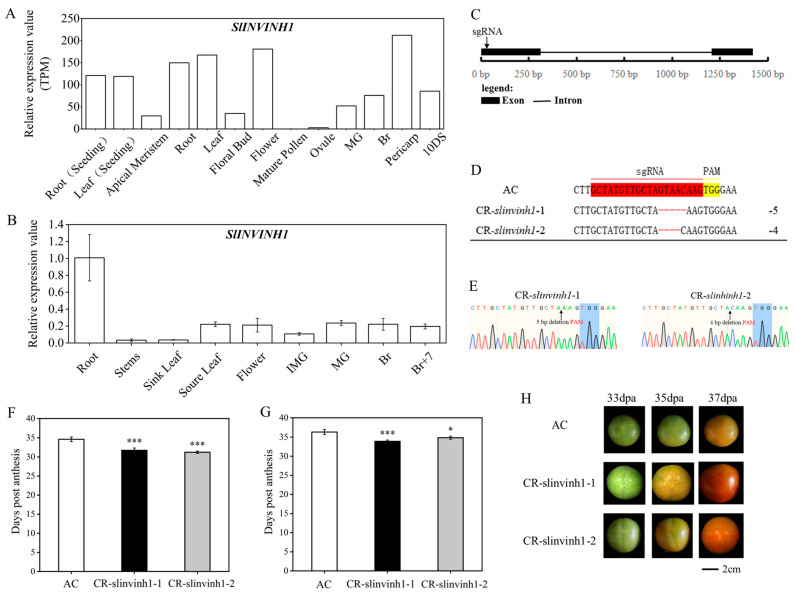
Knockout of *SlINVINH1* in tomato influenced the onset of fruit ripening. (**A**) Expression analysis of *SlINVINH1* in different tissues/organs of tomato. Data are from online transcriptome data. (**B**) qPCR analysis of *SlINVINH1* in different tissues/organs of tomato. Expression levels are presented as mean ± standard error of three biological replicates. (**C**) Gene structure of *SlINVINH1*. The position of the sgRNA designed to knock out *SlINVINH1* by CRISPR/Cas9 is indicated. (**D**) The gene editing patterns of the two homozygous CRISPR/Cas9 edited lines of *SlINVINH1*. (**E**) Details of the gene sequencing data of the two homozygous edited lines of *SlINVINH1*. (**F**,**G**) Analysis of the days required for the onset of fruit ripening in the CR-*slinvinh1* mutants growing in two independent glasshouses. Mean ± standard error is presented. N = 37, 41, 38, respectively, for AC, CR-*slinvinh1-1*, CR-*slinvinh1-2* in (**F**), and N = 54, 58, 46, respectively, for AC, CR-*slinvinh1-1*, *CR-slinvinh1-2* in (**G**). Statistical analysis was performed by *t*-test using IBM SPSS Statistics 25 software: *, *p* < 0.05; ***, *p* < 0.001. (**H**) Comparison of fruit color change between WT and the CR-*slinvinh1* mutants during fruit ripening. IMG, immature green; MG, mature green; Br, breaker; 10DS, 10-day seeds; AC, Alisa Craig; CR, CRISPR; dpa, days post anthesis.

When checking the online expression data of the cell wall invertase inhibitor gene, *SlINVINH1*, we found that it had high expression in the breaker (the onset of fruit ripening) stage and in the pericarp (Figure 1A), indicating its possible role in controlling fruit ripening. We further verified its high expression during fruit ripening through qPCR analysis (Figure 1B; Primers were listed in Table S1). Then, we knocked out *SlINVINH1* in tomato by using the CRISPR/Cas9 technique and obtained two independent effective homozygous gene edited lines, CR-*slinvinh1-1* and CR-*slinvinh1-2*, with 5 bp and 4 bp deletions, respectively, in the first exon of the gene coding region (Figure 1C–E). We investigated the number of days post anthesis required for the fruit to ripen in WT and the CR-*slinvinh1* mutants and found that knockout of *SlINVINH1* accelerated tomato fruit ripening by 2–3 days in a glasshouse (Figure 1F). We repeated the experiment in another glasshouse with similar environmental settings as the previous one and obtained similar result (Figure 1G,H). When the fruits of CR-*slinvinh1-1* and *CR-slinvinh1-2* turned an orange color, the fruit of WT was still green (Figure 1H). The above observations indicated that knockout of *SlINVINH1* accelerated tomato fruit ripening.

### 2.2. Analysis of Invertase Activity and Contents of Sugar, Chlorophyll and Carotenoid in the SlINVINH1 Knockout Lines During Ripening

**Figure 2:**
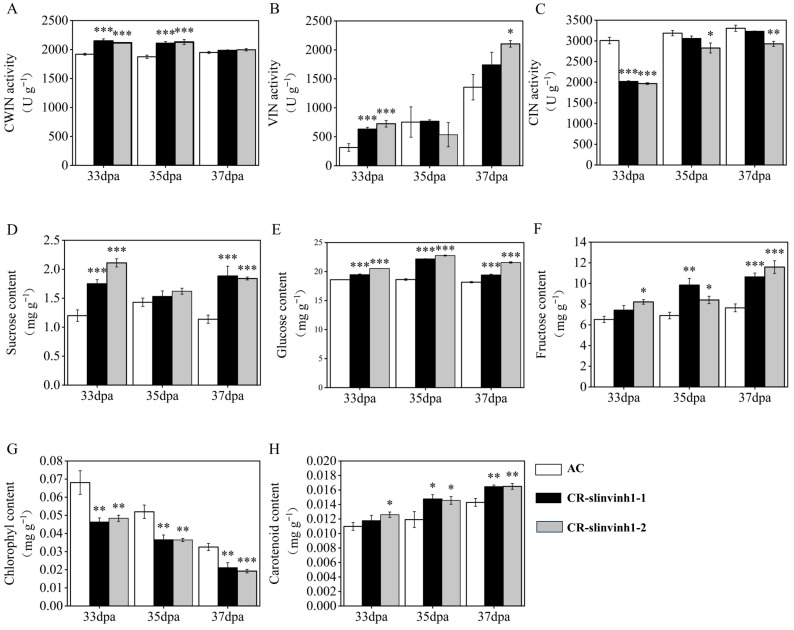
Analysis of invertase activities (**A**–**C**), contents of sugars (**D**–**F**), chlorophyll (**G**) and carotenoid (**H**) in CR-*slinvinh1* during fruit ripening. Data are presented as mean ± standard error of four biological replicates. Statistical analysis was performed by *t*-test using IBM SPSS Statistics 25 software: *, *p* < 0.05; **, *p* < 0.01; ***, *p* < 0.001. dpa, days post anthesis.

As *SlINVINH1* has been proven to interact with CWIN, regulating its activity at the post-translational level [13], we detected cell wall invertase activity in the CR-*slinvinh1* mutants during fruit ripening. It was found that the CWIN activity was significantly increased in the 33 and 35 dpa fruits, while remaining unchanged in the 37 dpa fruit, of both the two independent CR-*slinvinh1* mutants, compared with WT (Figure 2A). We also examined the activities of the other two kinds of invertases. The VIN activity was increased in the 33 and 37 dpa fruits, while remaining unchanged in the 35 dpa fruit, of both the two independent CR-*slinvinh1* mutants (Figure 2B). Unlike the two acid invertases, the alkaline/neutral CIN activity exhibited a reverse trend, decreasing particularly at 33 dpa during fruit ripening in the CR-*slinvinh1* mutants (Figure 2C).

As the alteration of invertase activity may influence sugar metabolism in plants, we further examined the change in sugar levels in the fruit of the CR-*slinvinh1* mutants during fruit ripening. The content of sucrose was significantly increased in the 33 and 37 dpa fruits of both the two CR-*slinvinh1* lines, while showing a nonsignificant increase in the 35 dpa fruit of the two mutants in comparison with WT (Figure 2D). Both the contents of glucose and fructose were significantly increased in the 33, 35 and 37 dpa fruits of the CR-*slinvinh1* mutants during fruit ripening (Figure 2E,F).

During the ripening process of tomato fruits, there is usually a decrease in chlorophyll content and an increase in carotenoid content within the fruit [1]. Hence, we also investigated the change in the contents of chlorophyll and carotenoid in the fruit of CR-*slinvinh1* mutants during fruit ripening. As expected, the chlorophyll content kept decreasing, while the carotenoid content kept increasing in the 33, 35, and 37 dpa fruits of both the two homozygous CR-*slinvinh1* mutants, in comparison to those of WT (Figure 2G,H).

### 2.3. Transcriptome Analysis of Fruit Samples from the SlINVINH1 Knockout Lines During Ripening

**Figure 3:**
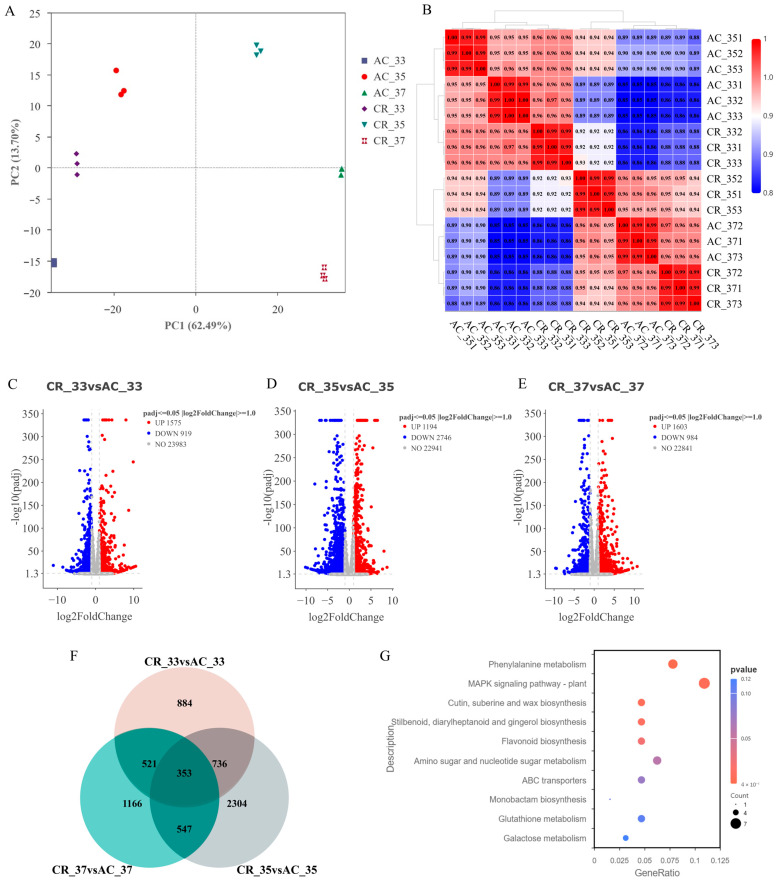
Transcriptome analysis of fruit samples from the CR-*slinvinh1-1* mutant during fruit ripening. (**A**) Principal component analysis of transcriptome data from the 33, 35, and 37 day-old fruit samples of WT and CR-*slinvinh1-1*, respectively. (**B**) Pearson correlation coefficient analysis of transcriptome data. (**C**–**E**) Volcano plots of differently expressed genes in fruit samples between WT and CR-*slinvinh1-1* at 33 (**C**), 35 (**D**), and 37 (**E**) dpa, respectively. (**F**) Venn diagram analysis for shared differentially expressed genes among 33, 35, and 37 dpa. (**G**) KEGG enrichment analysis of the common differentially expressed genes shared by 33, 35, and 37 dpa.

In order to investigate the mechanism of the CWIN inhibitor in regulating fruit ripening, we performed transcriptome sequencing analysis using the 33, 35 and 37 dpa fruit samples from the CR-*slinvinh1-1* mutant and WT. In total, 18 RNA samples were extracted and conducted for transcriptome sequencing (Table S2). After filtering the low-quality reads, a total of 124 Gb clean data were obtained with an average of 46.47 million clean reads per sample, over 97.75% of the Q30 and no less than 43.39% of the GC content (Table S2). The PCA and Pearson correlation coefficient analysis showed that the three biological replicates of each sample of CR-*slinvinh1-1* and WT had good consistency (Figure 3A,B), indicating the good quality and reliability of the transcriptome sequencing data.

To analyze the transcriptome difference between CR-*slinvinh1-1* and WT during fruit ripening, we first performed an analysis of the differentially expressed genes (DEGs). More than 2000 DEGs were identified for each time point of fruit ripening in CR-*slinvinh1-1* (Figure 3C–E). Gene Ontology (GO) enrichment analysis for the DEGs at each time point identified several biological processes related to cell wall and hydrolase activity, including cell wall, extracellular region, apoplast, transferase activity (transferring hexosyl/glycosyl groups), hydrolase activity (acting on glycosyl bonds, hydrolyzing O-glycosyl compounds), and glucosyltransferase activity, which are associated with the function of CWIN and its inhibitor (Figure S1A–C; Tables S3–S5). Kyoto Encyclopedia of Genes and Genomes (KEGG) enrichment analysis for the DEGs identified many pathways associated with fruit ripening and/or sugar metabolism, including plant hormone signal transduction, MAPK signaling pathway—plant, flavonoid biosynthesis, phenylpropanoid biosynthesis, photosynthesis, starch and sucrose metabolism, galactose metabolism, pentose and glucuronate interconversions (Figure S1D–F; Tables S6–S8). Some of these KEGG pathways have been recently reported to be involved in fruit ripening [18].

The number of DEGs between CR-*slinvinh1-1* and WT from 33, 35, and 37 dpa fruits were compared via Venn diagram analysis. As a result, 353 DEGs were found to be shared by the 33, 35, and 37 dpa fruits, and nearly two thirds (227 out of 353) of the DEGs encode transcription factors (Figure 3F; Table S9). We further conducted KEGG enrichment analysis for the shared DEGs, and identified a few important pathways related to fruit ripening, including phenylalanine metabolism, MAPK signaling pathway—plant, flavonoid biosynthesis (Figure 3G; Table S10).

### 2.4. Expression Analysis of Invertase and Inhibitor Genes in Fruits of SlINVINH1 Knockout Lines During Ripening

**Figure 4:**
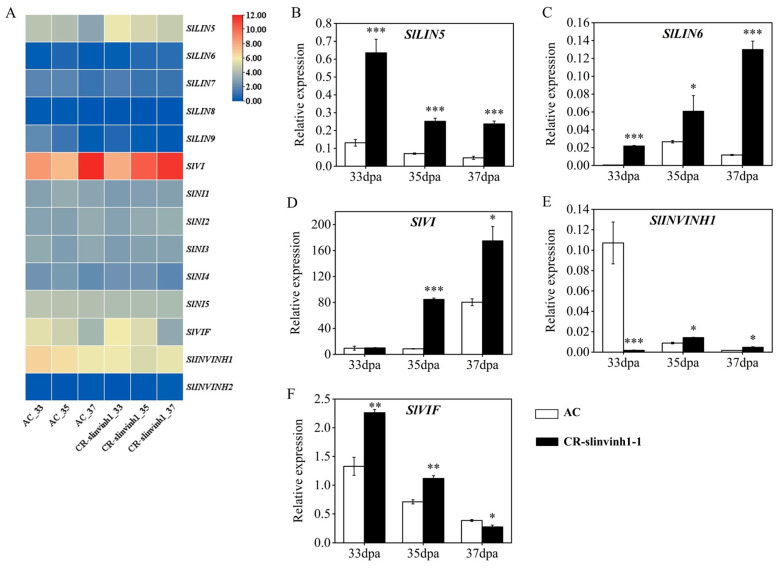
Expression analysis of invertase and its inhibitor genes in the fruit of CR-*slinvinh1-1* during fruit ripening. (**A**) Heatmap analysis of the expression of invertase and its inhibitor genes during fruit ripening. Expression values, presented as log_2_(FPKM+1), are from our transcriptome data. (**B**–**F**) qPCR verification of the expression of selected invertase and its inhibitor genes during fruit ripening. Expression levels are presented as mean ± standard error of three biological replicates. Statistical analysis was performed by *t*-test using IBM SPSS Statistics 25 software: *, *p* < 0.05; **, *p* < 0.01; ***, *p* < 0.001.

Since the invertase activities and sugar levels were altered in the *CR-slinvinh1-1* mutants during fruit ripening compared with WT (Figure 2), we investigated the expression patterns of related invertase and the inhibitor genes in fruits of CR-*slinvinh1-1* during ripening (Figure 4). Based on our transcriptome data, among the four CWIN genes (*SlLIN5*, *SlLIN6*, *SlLIN7*, *SlLIN8*) in tomato, *SlLIN5* was the major CWIN gene highly expressed during fruit ripening, and its transcript level was increased in all the 33, 35, 37 dpa fruits of CR-*slinvinh1-1* compared with WT (Figure 4A; Table S11), which may be responsible for the elevation in the CWIN activity (Figure 2A). For the two tomato VIN genes, *SlLIN9* was almost not expressed, while *SlVI* had very high expression, during fruit ripening. The transcript level of *SlVI* was largely increased in the 35 dpa fruits, and it was slightly decreased in the 33 and 37 dpa fruits, of CR-*slinvinh1-1* (Figure 4A; Table S11). All the CIN genes (*SlNI1*-*SlNI5*) had a few expression values during fruit ripening, with *SlNI5* exhibiting the highest transcript level among them. The transcript levels of all the CIN genes except *SlNI2* were slightly decreased across the fruit ripening stages in the fruit of CR-*slinvinh1-1* (Figure 4A; Table S11), in accordance with the decrease in CIN activity (Figure 2C). The transcript level of the sole vacuolar invertase inhibitor gene *SlVIF* was increased in the 33 and 35 dpa fruits and decreased in the 37 dpa fruit of CR-*slinvinh1-1* (Figure 4A; Table S11). For the two CWIN inhibitor genes, the transcript level of *SlINVINH1* was largely decreased in 33 and 35 dpa fruits and slightly decreased in the 37 dpa fruit of CR-*slinvinh1-1* in comparison to WT, whilst the transcript level of *SlINVINH2* was almost undetectable in the ripening stage fruit (Figure 4A; Table S11). The expression patterns of the major invertase and the inhibitor genes in our transcriptome data were further confirmed via qPCR analysis (Figure 4B–F).

### 2.5. SlINVINH1 Affects Fruit Ripening by Modulating the Expression of Pivotal Ripening-Related Marker Genes

**Figure 5:**
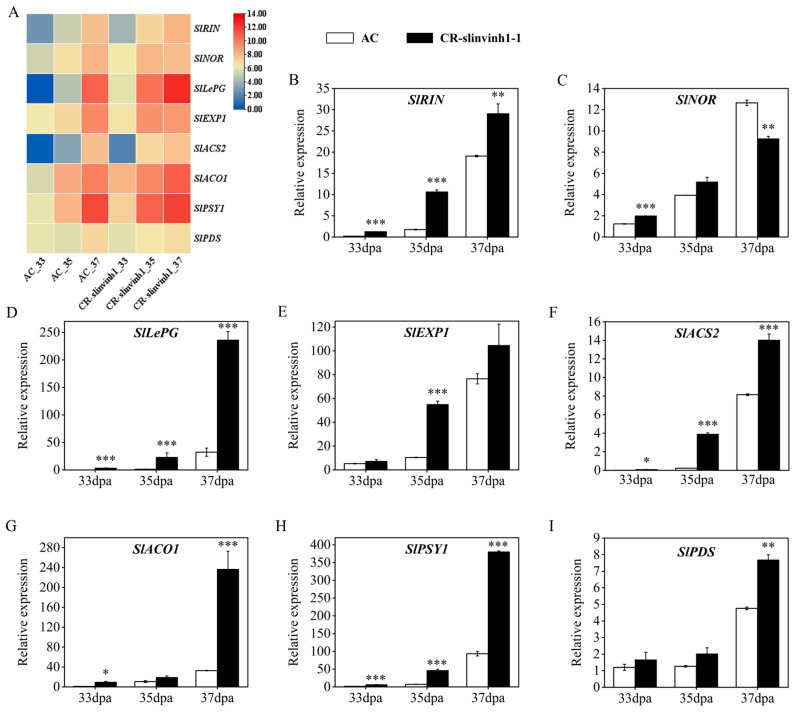
Expression analysis of key ripening-related marker genes in the fruit of CR-*slinvinh1-1* during fruit ripening. (**A**) Heatmap analysis of the expression of ripening-related marker genes during fruit ripening. Expression values, presented as log_2_(FPKM+1), are from our transcriptome data. (**B**–**I**) qPCR verification of the expression of ripening-related marker genes during fruit ripening. Expression levels are presented as mean ± standard error of three biological replicates. Statistical analysis was performed by *t*-test using IBM SPSS Statistics 25 software: *, *p* < 0.05; **, *p* < 0.01; ***, *p* < 0.001. *RIN*, *ripening inhibitor*; *NOR*, *non-ripening*; *LePG*, *polygalacturonase A*; *EXP1*, *expansin 1*; *ACS2*, *1-aminocyclopropane-1-carboxylate synthase 2*; *ACO1*, *1-aminocyclopropane-1-carboxylate oxidase*; *PSY1*, *phytoene synthase 1*; *PDS*, *phytoene desaturase*.

To better understand the mechanism of *SlINVINH1* in regulating fruit ripening, the transcript levels of a set of key ripening-related marker genes (*SlRIN*, *SlNOR*, *SlLePG*, *SlEXP1*, *SlACS2*, *SlACO1*, *SlPSY1*, and *SlPDS*) [19] were evaluated in CR-*slinvinh1-1* based on transcriptome data (Figure 5A; Table S11). The results indicated that the expression levels of these ripening marker genes were significantly influenced by *SlINVINH1*. In detail, the transcript levels of almost all these genes were largely increased at most time points of the ripening process in the fruits of CR-*slinvinh1-1* (Figure 5A; Table S11). Our qPCR analysis results further confirmed the expression patterns of these ripening-related marker genes in CR-*slinvinh1-1* during fruit ripening (Figure 5B–I). These results suggest *SlINVINH1* could negatively regulate fruit ripening by modulating the expression of essential ripening-related marker genes.

## 3. Discussion

CWIN inhibitors regulate the activity of CWINs at the posttranslational level, thus affecting sugar levels in plants, and have been revealed to play essential roles in the development of plant organs including the fruit, seed, leaves, tuber, and stress resistance [5,13,15,20,21,22]. In the current study, we discovered a novel role of CWIN inhibitors in regulating fruit ripening. We found the tomato CWIN inhibitor SlINVINH1 acts as a negative regulator in controlling the onset of fruit ripening, as knockout of the *SlINVINH1* gene through CRISPR/Cas9 technique accelerated the onset of tomato fruit ripening by approximately 2–3 days (Figure 1). The function of CWIN inhibitors in regulating the onset of fruit ripening has not been clearly reported previously, while once one VIN inhibitor was reported to have a role in controlling the onset of fruit ripening. In detail, Qin et al. (2016) showed that the tomato VIN inhibitor SlVIF positively influences fruit ripening, based on the findings that the overexpression of *SlVIF* accelerated the onset of tomato fruit ripening, whereas repression of *SlVIF* by RNA interference delayed fruit ripening [19]. Comparing our study with the previous one, we may conclude that the CWIN inhibitor SlINVINH1 and the VIN inhibitor SlVIF have opposite effects on tomato fruit ripening. We may wonder how the two types of invertase inhibitors exhibit adverse influence on tomato fruit ripening. Both the knockout of *SlINVINH1* gene in our study and the silencing of *SlVIF* gene increased the contents of glucose and fructose in tomato fruits during ripening. The former also increased the sucrose level, as did *SlVIF* overexpression, while the latter decreased the sucrose content [19]. It was previously reported that an external sucrose supply promoted color change in citrus and tomato fruits via affecting the synthesis of carotenoids [23,24]. Taken together, it may be speculated that the inverse effect on the onset of tomato fruit ripening from the knockout of *SlINVINH1* and the silencing of *SlVIF* may be due to their different influences on sucrose content, which requires further experimental validation in the future. Another possible reason for the inverse roles of SlINVINH1 and SlVIF in controlling the onset of fruit ripening may be the difference of their subcellular localizations. It would be of special significance to further unravel the authentic mechanisms mediated by the two types of invertase inhibitors in controlling the onset of fruit ripening in the future. In our study, knockout of *SlINVINH1* promoted the onset of fruit ripening with an increase in the contents of all the three major types of sugars (sucrose, glucose and fructose), which is in accordance with a very recent study that knocking out of *SlABF4* via CRISPR/Cas9 technique accelerated the onset of fruit ripening, along with the elevation in sucrose, glucose and fructose contents [18].

In this study, the transcript levels of several ripening related marker genes were largely increased in the fruit of the CR-*slinvinh1-1* mutant during fruit ripening (Figure 5; Table S11), which includes *SlRIN* encoding a MADS box transcription factor, a global regulator of tomato fruit ripening [19,25,26]. Previously through mutant analysis, expression analysis and protein-promoter binding assays, it was demonstrated that, during fruit ripening, RIN controls sugar metabolism by directly modulating the expression of genes involved in sucrose synthesis and degradation, which include invertase genes and their inhibitors genes [19]. Among these genes, *SlINVINH1* and *SlVIF* were the direct targets of SlRIN, while their expression levels were affected differently by SlRIN. Specifically, the transcript level of *SlINVINH1* was lower in the fruit of the *rin* mutant during fruit ripening, while that of *SlVIF* was higher [19], indicating that the roles of the two types of invertase inhibitor genes in regulating fruit ripening might be different, which is consistent with the findings in our study and in the previous report [19]. Notably, the transcript level of *SlRIN* was largely increased in the fruit of CR-*slinvinh1-1* during fruit ripening (Figure 5; Table S11), implying that SlINVINH1 may influence *SlRIN* expression via a possible negative feedback loop, which is converse to SlVIF as the expression of *SlRIN* was substantially reduced in the *SlVIF* RNAi tomatoes during fruit ripening. These findings again proved the inverse roles of SlINVINH1 and SlVIF in controlling fruit ripening. In the future, it would be significant to substantially unravel the complicated mechanisms mediated by the interactions between SlINVINH1, SlVIF, SlRIN, and possible sugar signaling in influencing tomato fruit ripening.

## 4. Materials and Methods

### 4.1. Plant Materials and Growth Conditions

All tomato plants used in this study were Alisa Craig and were grown in environmentally controlled glasshouses with a photoperiod of approximately 16 h light at 25 °C and 8 h dark at 22 °C. Different tissues/organs from different developmental stages of the tomato plants were sampled, frozen in liquid nitrogen, and stored in a freezer at −80 °C for later RNA extraction, transcriptome sequencing and/or determination of physiological and biochemical traits.

### 4.2. RNA Extraction, cDNA Synthesis and qPCR Analysis

Total RNA was extracted using the FastPure universal plant total RNA isolation kit (#RC411-01, Vazyme, Nanjing, China), according to the manufacturer’s instructions. The concentration and purity of RNA were determined using the NanoDrop Lite (Thermo Scientific, Waltham, MA, USA), and the integrity of RNA was detected by agarose gel electrophoresis. cDNA was synthesized using total RNA (1 μg for each sample) via the ToloScript All-in-one RT EasyMix for qPCR (#22107-01, TOLOBIO, Shanghai, China), which includes a gDNA elimination step. Synthesized cDNA was diluted 10-fold and used as the template for subsequent qPCR (quantitative polymerase chain reaction) analysis. qPCR was performed using the 2 × Q3 SYBR qPCR Master mix (Universal, #22204-01, TOLOBIO, Shanghai, China) on the qPCR platform LightCycler 480II (Roche, Basel, Switzerland). Three biological replicates and three technical replicates were performed for qPCR analysis, and the 2^−ΔΔCT^ algorithm [27] was used for calculating the relative gene expression levels. *SlSAND* was used as the reference gene to normalize the gene expression values [14]. The primers utilized in the qPCR analysis are listed in Table S1.

### 4.3. Generation of CRISPR/Cas9 Mediated Gene Knockout Mutant Lines

Based on the gene ID of *SlINVINH1*, the sgRNA sequence (GCTATGTTGCTAGTAACAAG) for constructing the CRISPR/Cas9 vector was designed using free online software (http://crispr.dfci.harvard.edu; http://skl.scau.edu.cn). Subsequently, CRISPR/Cas9 gene knockout vector and tomato genetic transformation were performed with help from the Shanghai Zhishuo Biotechnology Co., Ltd. (Shanghai, China). After the recombinant vector was verified to be correct by Sanger sequencing, it was transformed into *Agrobacterium*. Tomato seeds were sterilized and then inoculated on 1/2 MS medium to cultivate sterile seedlings. After 6 days of cultivation, the cotyledon explants were taken for pre-culture for 1 day. Meanwhile, *Agrobacterium* containing the recombinant plasmid was cultured to the logarithmic phase. After centrifugation and suspension of the *Agrobacterium* containing the recombinant plasmid, it was used to infect the explants for 4–5 min. Subsequently, co-culture, induction of callus and adventitious buds on selection medium with gradient concentrations of zeatin riboside and rooting on RS medium were carried out. Finally, the plantlets generated were transplanted into pots for further cultivation. Primers covering the sgRNA were designed based on the *SlINVINH1* genomic sequence, and PCR amplification was performed using the DNA of the obtained transgenic plants as the template. The editing mode of the target gene was detected by Sanger sequencing. The seeds of the plants with effective gene editing were collected, propagated, segregated and PCR identified, and finally, Cas9-free homozygous edited mutant lines were obtained.

### 4.4. Fruit Ripening-Related Phenotypic Analysis and Fruit Sample Preparation

Each flower of the tomato plants was marked on the day of flowering, and the flowering date was recorded. Each tomato fruit that was turning color (breaker stage) was also marked on the day of color change, and the color change date was recorded. Based on the flowering date and the color change date, the number of days required for each tomato fruit to turn color was obtained. For the transgenic background and the obtained knockout mutant lines, the color change data of more than 30 fruits (see Figure 1 caption for detailed numbers) were recorded and statistically analyzed. Only the fruits of the first and second trusses (clusters) of each tomato plant were used for analyzing the number of days required for turning color. The photos of the representative fruits during the color change period were taken with a Canon single-lens reflex camera.

For 33, 35 and 37 dpa fruit sampling, three tomato fruits from the same position of the same order truss of three different tomato plants were taken as one biological replicate. In total, four biological replicates of fruit samples were collected. For each biological replicate, three tomato fruits were immediately cut into approximately one cubic centimeter pieces after sampling, frozen in liquid nitrogen, and stored in a freezer at −80 °C. Subsequently, the fruit sample of each biological replicate was ground into uniform powder using a mortar with liquid nitrogen and then transferred into RNase-free 50-mL tubes and again stored at −80 °C, before being used for RNA extraction/transcriptome sequencing and/or determination of physiological and biochemical traits.

### 4.5. Determination of Invertase Activity, Sugar Levels and the Contents of Chlorophyll and Carotenoid

Detection of the activities of CWIN (#BC4325) [28], VIN (#BC0565) [29] and CIN (#BC0575) [29] and the contents of sucrose (#BC2460) [28], glucose (#BC2500) [28], fructose (#BC2455) [30], chlorophyll (#BC0990) [31], and carotenoids (#BC4330) [32] were performed as previously reported in the literature, using their respective ELISA kits from the Beijing Solarbio Science & Technology Co., Ltd. (Beijing, China). Plant tissue extraction solutions were prepared following the manufacturer’s instructions. For each biological replicate, approximately 0.1 g frozen fresh sample power was used for the detection of one item using each ELISA kitfrom the Beijing Solarbio Science & Technology Co., Ltd. (Beijing, China). The absorbance was recorded using a microplate reader (Tecan Spark, Männedorf, Switzerland). For detection of the activities of CWIN, VIN and CIN, the absorbance of sample reactions was monitored at 540 nm, and drawing of standard curves was included in the protocols. For sucrose and fructose, the absorbance of sample reactions was monitored at 480 nm, while that of glucose was monitored at 505 nm. For chlorophyll, the absorbance of sample reactions was monitored at 645 and 663 nm, while that of carotenoids was monitored at 470, 646 and 663 nm. For detailed protocols, please visit the official website (https://www.solarbio.com) and search using the Cat No. of the ELISA kits for more information.

### 4.6. Transcriptome Analysis

Transcriptome analysis was performed based on the similar methods described previously [33]. In brief, fruit samples at 33, 35 and 37 dpa from AC and *CR-slinvinh1-1*, respectively, were collected and sent to the Beijing Novogene Co., Ltd. (Beijing, China) for transcriptome sequencing. First, RNA samples were built into cDNA libraries before being sent to DNBSEQ-T7 for sequencing. Raw data in FASTQ format were firstly processed through the Fastp software (version 0.19.4) [34], and Q20, Q30 and GC content of the clean data generated were calculated at the same time. Subsequently, clean reads were aligned against the reference tomato genome (version SL4.0, https://solgenomics.net/organism/Solanum_lycopersicum/genome, accessed on 11 October 2025) using the Hisat2 (version 2.2.1) [35]. The mapped reads of each sample were assembled by StringTie (version 2.2.3) [36] in a reference-based approach. FeatureCounts (version 2.0.6) was used to count the reads numbers mapped to each gene. Then, the Fragments Per Kilobase of exon per Million fragments mapped (FPKM) value of each gene were calculated based on the length of the gene and reads count mapped to this gene. Differential expression analysis for two conditions/groups was performed using the DESeq2 R package (version 1.42.0) with the threshold of significant differential expression: padj <= 0.05 & |log2(foldchange)| >= 1. Enrichment analyses, including Gene Ontology (GO) enrichment analysis and the Kyoto Encyclopedia of Genes and Genomes (KEGG) pathway enrichment analysis, were implemented using the clusterProfiler (version 4.8.1). Heatmap analysis of the gene expression values was conducted using the TBtools software (version 2.372) [37].

### 4.7. Statistical Analysis

Experimental data generated in this study were processed firstly using Microsoft Excel 2021. The statistical significance of the data was analyzed using IBM SPSS Statistics 25 software (*t*-test), and relevant graphs were drawn using Origin 2024. The experimental data in the figures are presented as the mean ± standard error, with the significance levels shown as *, *p* < 0.05; **, *p* < 0.01; ***, *p* < 0.001.

## 5. Conclusions

In conclusion, this study revealed a novel role of the plant CWIN inhibitor in regulating fruit ripening. The tomato CWIN inhibitor SlINVINH1 acts as a negative regulator in controlling the onset of fruit ripening through modulating sugar metabolism, the degradation/synthesis of chlorophyll and carotenoids, and the expression of ripening-related marker genes. In the future, the *SlINVINH1* gene could be utilized to genetically improve the tomato fruit ripening process and fruit sweetness simultaneously by manipulating its expression, which could have potential significant value for tomato breeding.

## Data Availability

The original contributions presented in this study are included in the article/Supplementary Material. Further inquiries can be directed to the corresponding authors.

## Supplementary Materials

The following supporting information can be downloaded at https://www.mdpi.com/article/10.3390/plants15060942/s1, Figure S1: GO (A–C) and KEGG (D–F) enrichment analyses of the differentially expressed genes at 33, 35, and 37 dpa, respectively, between WT and CR-*slinvinh1-1*; Table S1: Primers used in this study; Table S2: Quality assessment of transcriptome sequencing data; Table S3: GO enrichment analysis of the DEGs in 33 dpa fruits between CR-*slinvinh1* and WT; Table S4: GO enrichment analysis of the DEGs in 35 dpa fruits between CR-*slinvinh1* and WT; Table S5: GO enrichment analysis of the DEGs in 37 dpa fruits between CR-*slinvinh1* and WT; Table S6: KEGG enrichment analysis of the DEGs in 33 dpa fruits between CR-*slinvinh1* and WT; Table S7: KEGG enrichment analysis of the DEGs in 35 dpa fruits between CR-*slinvinh1* and WT; Table S8: KEGG enrichment analysis of the DEGs in 37 dpa fruits between CR-*slinvinh1* and WT; Table S9: Details of shared DEGs at 33, 35 and 37 dpa; Table S10: KEGG enrichment analysis of shared DEGs at 33, 35 and 37 dpa; Table S11: The average expression value (FPKM) of invertase, invertase inhibitor and ripening related marker genes.

## Abbreviations

The following abbreviations are used in this manuscript:

AC	Alisa Craig
Br	Breaker
CIN	Cytoplasmic invertase
CRISPR (CR)	Clustered Regularly Interspaced Short Palindromic Repeats
CWIN	Cell wall invertase
DEG	Differentially expressed gene
dpa	Days post anthesis
FPKM	Fragments Per Kilobase of exon model per Million mapped fragments
GO	Gene Ontology
IMG	Immature green
INVINH	Invertase inhibitor
KEGG	Kyoto Encyclopedia of Genes and Genomes
MG	Mature green
RIN	Ripening inhibitor
sgRNA	Single guide ribonucleic acid
VIN	Vacuolar invertase
