# Supplementary material for: Cell Wall Invertase Inhibitor SlINVINH1 Acts as a Negative Regulator in Fruit Ripening of Tomato
Source: Plants (Basel). 2026 Mar 19;15(6):942. doi: 10.3390/plants15060942 (PMC13029962; doi:10.3390/plants15060942)
Supplement: Supplementary file 1 [file plants-15-00942-s001.zip › Supplemental Figure 2026.02.09.pdf]

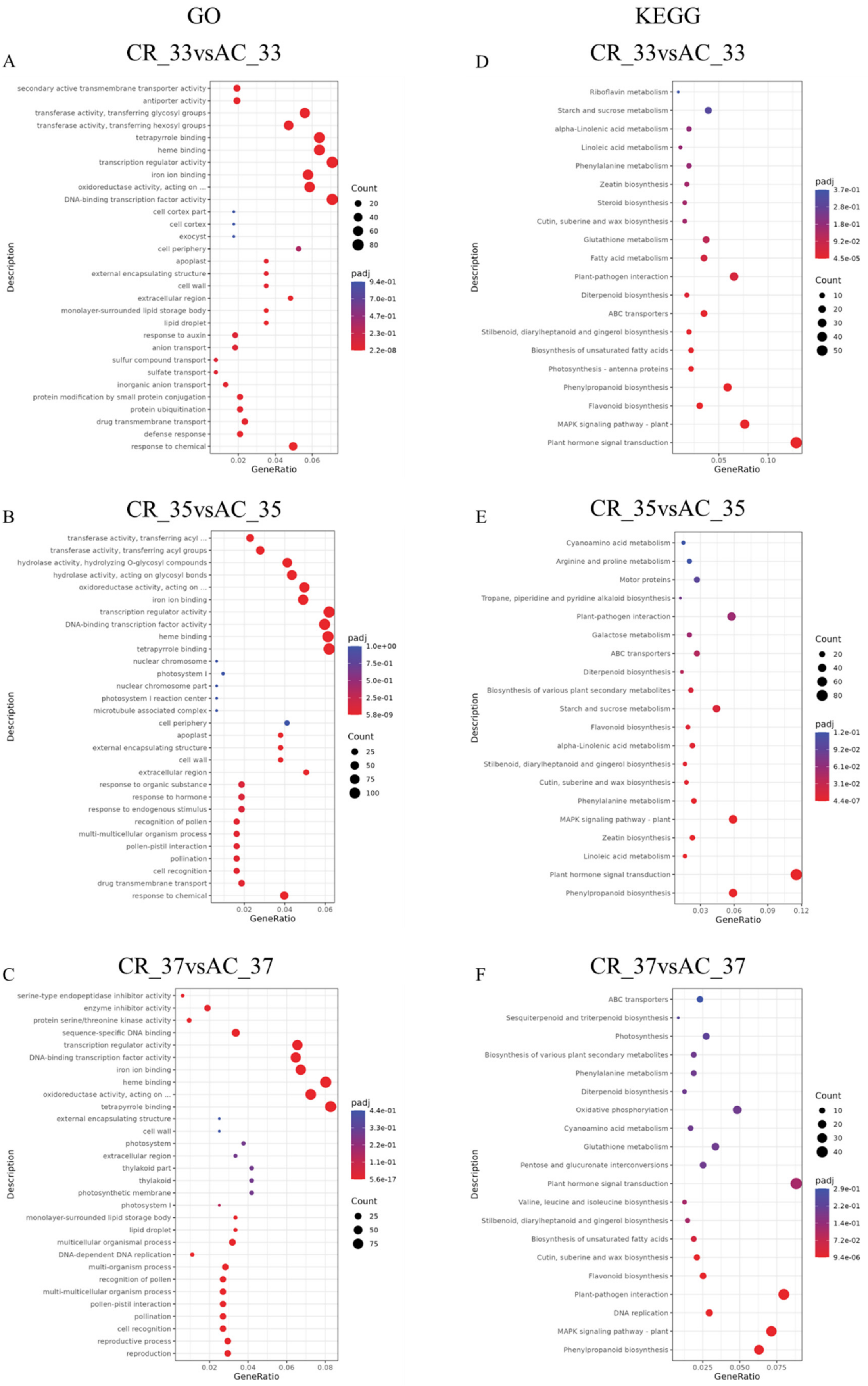

Figure S1. GO (A-C) and KEGG (D-F) enrichment analyses of the differentially

expressed genes at 33, 35, and 37 dpa, respectively between WT and CR-*slinvinh1-1*.
